# TET2 mutations contribute to adverse prognosis in acute myeloid leukemia (AML): results from a comprehensive analysis of 502 AML cases and the Beat AML public database

**DOI:** 10.1007/s10238-024-01297-0

**Published:** 2024-02-13

**Authors:** Xin’an Pan, Yingjun Chang, Guorui Ruan, Songhai Zhou, Hao Jiang, Qian Jiang, Xiaojun Huang, Xiao-Su Zhao

**Affiliations:** 1 Peking University People’s Hospital, Peking University Institute of Hematology, National Clinical Research Center for Hematologic Disease, Beijing Key Laboratory of Hematopoietic Stem Cell Transplantation, No. 11 Xizhimen South Street, Beijing, 100044 China; 2 Peking-Tsinghua Center for Life Sciences, Beijing, 100044 China; 3 Research Unit of Key Technique for Diagnosis and Treatments of Hematologic Malignancies, Chinese Academy of Medical Sciences, 2019RU029, Beijing, China

**Keywords:** Acute myeloid leukemia, TET2 mutations, Prognosis, AML Beat database, RNA sequencing, Bioinformatics analysis

## Abstract

Despite the high incidence of tet methylcytosine dioxygenase 2 (TET2) mutations in acute myeloid leukemia (AML), the prognostic implications of these mutations in three AML risk groups based on the 2022 ELN AML risk classification are still unclear. A total of 502 consecutive de novo AML patients who had next-generation sequencing data available between March 2011 and July 2021 at the Peking University Institute of Hematology were enrolled in this study. Univariate and multivariate Cox regression analyses were performed to explore the prognostic impact of TET2 mutations in the above cohort and the Beat AML cohort. Of the 502 total AML patients, 76 (15.1%) carried TET2 mutations. Multivariate analysis revealed TET2 mutations as independent risk factor for overall survival (OS) in both the total AML cohort (OR = 1.649, *p* = 0.009) and in the 2022 ELN intermediate-risk cohort (HR = 1.967, *p* = 0.05). Analysis of RNA-seq data from the Beat AML study revealed 1042 differentially expressed genes (DEGs) between the TET2-mutant and TET2 wild-type groups. The results of enrichment analysis indicated the DEGs to be notably enriched in categories related to the PI3K-Akt signaling pathway. Collectively, our findings indicate that mutations in TET2 are prognostically disadvantageous in AML patients. Assessment of TET2 mutational status contributes to the stratification of intermediate-risk AML patients. Multiple genes and pathways of potential therapeutic relevance may be differentially modulated by TET2 mutations in AML.

**Supplementary Information:**

The online version contains supplementary material available at 10.1007/s10238-024-01297-0.

## Introduction

Cutting-edge technologies, including genome-wide whole-exome sequencing (WES), have unveiled a multitude of recurrent mutations in hematologic neoplasms [1]. These genetic alterations have important implications for diagnosis and risk stratification of acute myeloid leukemia (AML) patients [2]. Similar to DNA methyltransferase 3 alpha (DNMT3A) and ten-eleven translocation 2 (TET2) dioxygenase, ASXL transcriptional regulator 1 (ASXL1) is a core epigenetic regulator of myeloid neoplasms involved in cytosine demethylation and immune homeostasis. TET2 can oxidize 5-methylcytosine (5mC) to generate 5-hydroxymethylcytosine (5hmC), hence promoting DNA demethylation [3, 4]. TET2 mutations are closely related to a reduction in 5hmC content, and it has been acknowledged that TET2 mutations may serve as a promising diagnostic and prognostic biomarker for hematopoietic malignancies [4]. TET2 expression is elevated in hematopoietic stem and progenitor cells (HSPCs) [5], and TET2 is broadly considered to be a gene with tumor-suppressor function. Deletion of TET2 causes hematologic malignancies in mice [6]. The initial predominant advantage of TET2 in CD34+ CD38+ cells is that TET2 also contributes to monocyte expansion, which implicates TET2 in lineage commitment [7]. Inactivating mutations in TET2 frequently occur in clonal hematopoiesis of indeterminate potential (CHIP) and endow a hematopoietic stem cell advantage [8–10].

Despite having a pivotal role in hematopoiesis and exhibiting a relatively high mutation frequency (12–34%) in AML patients [11], the prognostic effect of TET2 mutational status in myeloid malignancies continues to be a matter of debate. Early research revealed that AML patients with TET2 mutations had poorer outcomes than patients with wild-type TET2 [12–14], and mutated TET2 has also been shown to confer adverse survival in chronic myeloid leukemia (CML) patients [15]. However, some cohort studies have shown that TET2 mutations do not predict worse overall survival (OS) in AML and myeloproliferative neoplasm (MPN) patients [16–18]. Furthermore, TET2 mutations can synergize with other driver mutations, such as FLT3-ITD, to induce AML [19]. With the aid of novel drugs, including hypomethylating agents (HMAs), the clinical significance of TET2 mutations requires further investigation.

Therefore, our study aimed to evaluate the prognostic significance of TET2 mutations in 502 intensively treated AML patients stratified by risk level according to the 2022 European LeukemiaNet (ELN) classification system. In addition, we analyzed RNA sequencing (RNA-Seq) data from the Beat AML cohort [20] to identify core pathways and genes associated with TET2 mutations. Our study is expected to provide additional insights into the role of TET2 mutations in AML and to facilitate development of novel DNA methylation-targeted therapeutics.

## Materials and methods

### Patients and samples

We enrolled 502 consecutive newly diagnosed AML patients with next-generation sequencing (NGS) profiling data between March 2011 and July 2021 from the Peking University Institute of Hematology (Peking cohort). Bone marrow mononuclear cells were isolated by Ficoll-Hypaque density gradient centrifugation (Solarbio Science and Tech., Beijing, China). DNA extraction was performed according to the manufacturer’s protocol using DNAzol kits (Invitrogen, Carlsbad, CA, USA). Clinical information and related RNA-seq mutation data from 582 AML patients were downloaded from the Beat AML study (http://www.vizome.org). The GSE32246 dataset, which contains both TET2 mutation data and RNA-seq data, was obtained from the GEO (Gene Expression Omnibus) database (https://www.ncbi.nlm.nih.gov/geo/). The research was authorized by the Ethics Committee of the Peking University People’s Hospital and adhered to the principles of the Declaration of Helsinki.

### Chemotherapy and transplantation procedures

The initial one to two cycles of induction therapy encompassed the following regimens: (i) a combination of cytarabine (administered at 100 mg/m^2^ over a period of 7 days) with either idarubicin (dosage of 10 mg/m^2^ per day for a duration of 3 days) or daunorubicin (45 mg/m^2^ daily for 3 days); (ii) a regimen comprising homoharringtonine (2 mg/m^2^ daily for 7 days), aclacinomycin (20 mg daily for 7 days), and cytarabine (100 mg/m^2^ for 7 days); or (iii) a protocol involving cytarabine (10 mg/m^2^ bidaily for 14 days), aclarubicin (20 mg daily for 4 days), and granulocyte colony-stimulating factor (G-CSF, 300 mg daily for 14 days). Patients who achieved histological complete remission (CR) received consolidation therapy. Subsequent to the second consolidation phase, patients and their attending physicians were informed of the merits and drawbacks of an allogeneic transplant versus continued consolidation chemotherapy. Those opting for a solely chemotherapeutic approach underwent four additional consolidation cycles, which included two cycles of intermediate-dose cytarabine (2 g/m^2^ every 12 h for 3 days) and two cycles of cytarabine (100 mg/m^2^ daily for 7 days) in conjunction with either daunorubicin (45 mg/m^2^ daily), idarubicin (10 mg/m^2^ daily), or mitoxantrone (8 mg/m^2^ daily), each administered over a period of 3 days. Patients in remission (NR) were switched to the FLAG protocol (fludarabine + Ara-C + G-CSF). Allogenic hematopoietic stem cell transplantation (allo-HSCT) procedures include conditioning protocols, stem cell harvesting, prevention of acute graft-versus-host disease, and supportive care. The chemotherapy and transplantation regimens have been described in detail by our research group in previously published reports [21, 22].

### High-depth targeted next-generation sequencing

High-depth targeted next-generation sequencing of 99 myeloid tumor-associated genes (Supplementary Data1_01.xlsx) was conducted on samples obtained from 502 AML patients at the time of initial diagnosis. A customized hematologic tumor panel (Twist Bioscience, USA) was used to identify targeted AML genes via use of genomic DNA. The captured DNA libraries were sequenced using the Illumina platform (Illumina, USA), with 150 bp paired-end reads generated after quality control.

### Bioinformatics analysis of the Beat AML database

Analysis of differentially expressed genes (DEGs) between TET2-mutant and non-TET2-mutant groups was performed by the “limma” R package. DEGs were identified based on an absolute log fold change (FC) of ≥ 2 and an FDR < 0.05. GO term enrichment and KEGG pathway analyses were carried out using the R packages “GAGE” and “Pathview.” A protein‒protein interaction (PPI) network was created with Cytoscape software. DEG-encoded proteins with a comprehensive rating > 0.4 were considered significant. We utilized the molecular complex detection (MCODE) plugin available in Cytoscape for module identification.

### End points and statistical analysis

The primary endpoint was overall survival (OS). The secondary endpoints were event-free survival (EFS) and the cumulative incidence of relapse (CIR). We measured CIR, EFS, and OS from the date of initial diagnosis. The events considered for CIR and OS were relapse and death, respectively. For EFS, we included relapse and death due to any cause. We created survival curves for EFS and OS using the Kaplan‒Meier method and compared differences between groups through log-rank analysis. To calculate CIR, we used Gray’s test for competing risk analysis with nonrelapse mortality. A two-sided P value of less than 0.05 was considered to indicate a statistically significant difference. All the analyses were conducted using R software 4.0.3.

## Results

### Patient characteristics

**Fig. 1:**
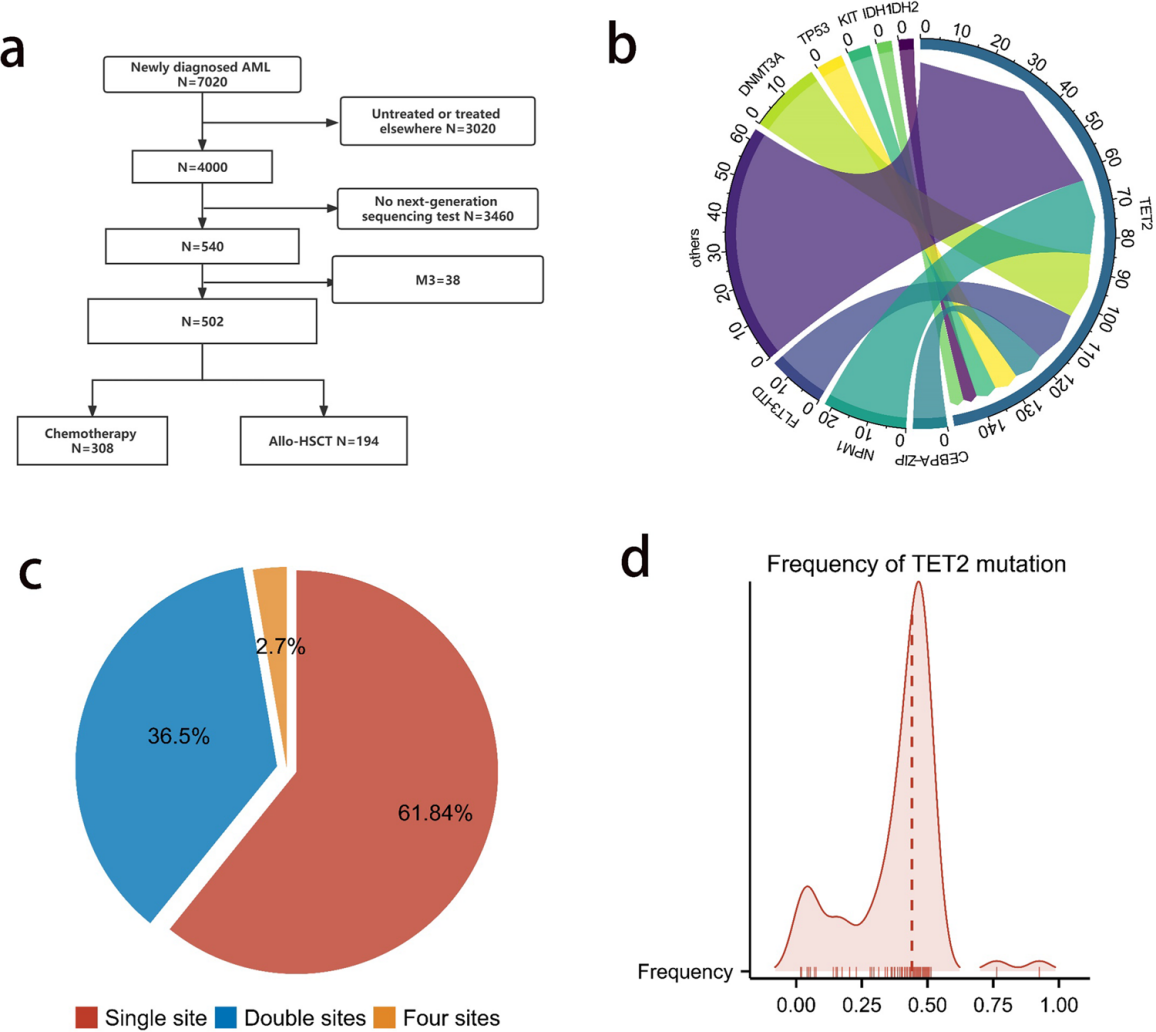
**a** The process of patient recruitment and cohort assignment in the Peking cohort. **b** The commutation status observed among patients in the Peking cohort. **c** The status of TET2 mutation sites within patients from the Peking cohort. **d** The frequency of TET2 mutations among AML patients within the Peking cohort. Others: mutations in ASXL1, BCOR, SRSF2, STAG2, U2AF1, EZH2, RUNX1, SF3B1, and ZRSR2

A total of 7020 patients with newly diagnosed AML were enrolled from February 2011 to August 2021 at our institute. Our study enrolled participants who met the following eligibility criteria: (1) received induction or consolidation chemotherapy at our hospital; (2) received complete morphology, immunology, and cytogenetic and molecular (MICM) assessment, including NGS tests, at our hospital; and (3) non-APL (Fig. 1a). After excluding 6518 individuals who did not meet the inclusion criteria for AML, we ultimately selected 502 patients, among whom 76 (15.1%) carried TET2 mutations. Consistent with the findings of prior research, there was a greater age distribution in the TET2 mutation group than in the WT TET2 group (55.0 vs. 46.0 years, *p* < 0.001; Table 1). A lower transplantation rate was recorded for the 76 patients harboring TET2 mutations than for those with TET2-WT status (27.6% vs. 40.6%, respectively; *p* = 0.044). There were no significant differences between the two cohorts in terms of sex, WBC count, hemoglobin level, platelet count, French–American–British (FAB) type, or bone marrow blast at diagnosis (all *p* > 0.05). Comparison of the incidence of AML-related mutations revealed that the incidence of concomitant ASXL1 and SRSF2 mutations was greater in the TET2-mutation group than in the TET2-WT group (21.1% vs. 9.9%, *p* = 0.009; 9.2% vs. 2.8%, *p* = 0.015; Supplementary Table S1). We further subdivided the 76 TET2+ AML patients into two groups based on the median mutation frequency and examined 19 types of AML-related mutations but found no significant difference in occurrence rates according to mutational burden (all *p* > 0.05; Supplementary Table S2).

**Table 1:** Patient characteristics of the Peking cohort categorized by TET2 mutation status

Characteristic	Category	TET2 wild-type	TET2 mutation	*P* value
N		426	76	
Sex, *n* (%)	Female	210 (49.3)	30 (39.5)	0.146
	Male	216 (50.7)	46 (60.5)	
FAB type, *n* (%)	M0	3 (0.7)	0 (0)	0.059
	M1	6 (1.4)	3 (3.9)	
	M2	271 (63.6)	49 (64.5)	
	M4	92 (21.6)	18 (23.7)	
	M5	54 (12.7)	6 (7.9)	
Cytogenetic risk stratification, *n* (%)	Favorable	82 (19.2)	9 (11.8)	0.222
	Intermediate	279 (65.5)	57 (75)	
	Adverse	65 (15.3)	10 (13.2)	
2022 ELN risk classification, *n* (%)	Favorable	199 (46.7)	32 (42.1)	0.618
	Intermediate	107 (25.1)	23 (30.3)	
	Adverse	120 (28.2)	21 (27.6)	
Allo-HSCT, *n* (%)	No	253 (59.4)	55 (72.4)	0.044
	Yes	173 (40.6)	21 (27.6)	
Age(years), median (IQR)		46 (32, 57)	55 (41, 64.5)	< 0.001
Bone marrow blast percentage, median (IQR)		54 (35, 70.5)	55.25 (33.12, 73.88)	0.492
WBC(× 10^9^/L), median (IQR)		10.86 (3.19, 35.44)	13 (3.88, 41.91)	0.357
Hemoglobin(g/L), median (IQR)		83 (63.25, 104)	89 (68, 112)	0.104
Platelets(× 10^9^/L), median (IQR)		52 (28, 97)	46.5 (21.75, 88.25)	0.252

*Allo-HSCT* allogenic hematopoietic stem cell transplantation, *FAB* French–American–British classification, *WBC* white blood cell, *WT* wild type, *Mut* mutation, *IQR* interquartile range

### Genomic analysis and basic information of patients with TET2-mutated AML

Among the 76 patients in the Peking cohort who carried TET2 mutations, the most prevalent commutation was in NPM1 (n = 22, 29.0%), followed by DNMT3A (n = 19, 25.0%), FLT3-ITD (n = 15, 19.7%), WT1 (n = 10, 13.2%), and CEBPA-ZIP (n = 9, 11.84%) (Fig. 1b). Moreover, 47 of those patients (61.8%) had single-locus TET2 mutations, while 27 patients (36.5%) had double TET2 mutation sites (Fig. 1c). The median frequency of TET2 mutation was 0.44 (IQR: 0.321–0.479) (Fig. 1d). We downloaded clinical and mutation data for 561 AML patients in the beat AML database and identified 65 patients (12%) with TET2 mutations (Supplementary Figure S1a). The mutation types included splice, truncating, and missense mutations spanning the entire gene (Supplementary Figure S1b).

### TET2 mutations predict lower OS in AML patients

**Fig. 2:**
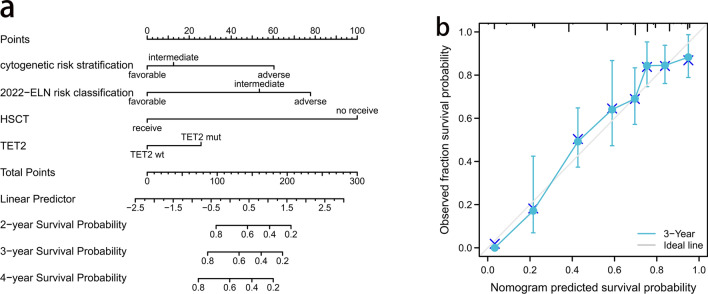
**a** Nomogram for all AML patients in the Peking cohort. A vertical line extending upward toward the “points” bar was created for point calculation. Then, depending on the resulting sum, a vertical line was projected downward from the “Total Points” line to determine OS. **b** Calibration curve for predicting survival probability in all AML patients in the Peking cohort. The gray line indicates that the nomogram survival probability equals the observed survival probability. The actual calibration is represented by the blue line. For each point along the calibration curve, the median is symbolized by circles, the mean is marked by “X,” and the 95% confidence intervals are illustrated for each point

The association of each covariate with OS across the entirety of the Peking cohort was examined through univariate analysis. As indicated in Table 2, in addition to allo-HSCT, cytogenetic risk stratification, the 2022 ELN risk classification, and TET2 mutation status correlated negatively with OS (all *p* < 0.01). Covariates with P values < 0.1 in univariate analysis were incorporated into a multivariate model. This analysis showed that TET2 mutations independently predicted shorter OS in AML patients in the Peking cohort (*p* = 0.009) (Table 2). Similar results were obtained via univariate and multivariate analyses of AML patients from the Beat AML database (Supplementary Table S3, *p* < 0.001). The four variables with *p* values < 0.05 in multivariate Cox analysis were eventually entered into the nomogram (Fig. 2a). All points corresponding to the 4 variables were summed to assess individual probabilities of 2-, 3-, and 4-year OS. The model exhibited good discrimination, with a C-index of 0.78 (95% confidence interval (CI), 0.76–0.80). The calibration curves for predicting patient OS at 3 years demonstrated a strong correlation between the predicted values obtained from the nomogram and the observed values (Fig. 2b).

**Table 2:** Univariate and multivariate analyses of OS in entire cohort of AML patients in the Peking cohort

Characteristics	Total(N)	Univariate analysis		Multivariate analysis	
		Hazard ratio (95% CI)	*P* value	Hazard ratio (95% CI)	*P* value
Sex	502		0.087		
Female	240	Reference		Reference	
Male	262	1.300 (0.961–1.759)	0.088	1.222 (0.896–1.666)	0.205
FAB type	502		0.616		
M0	3	Reference			
M1	9	0.346 (0.049–2.460)	0.289		
M2	320	0.589 (0.145–2.388)	0.459		
M4	110	0.673 (0.162–2.788)	0.585		
M5	59	0.690 (0.163–2.924)	0.615		
M7	1	2.933 (0.265–32.504)	0.381		
Cytogenetic risk stratification	502		** < 0.001**		
Favorable	91	Reference		Reference	
Intermediate	336	2.176 (1.248–3.795)	**0.006**	1.354 (0.709–2.586)	0.358
Adverse	75	6.468 (3.549–11.786)	** < 0.001**	3.453 (1.580–7.549)	**0.002**
2022 ELN risk classification	502		** < 0.001**		
Favorable	231	Reference		Reference	
Intermediate	130	2.140 (1.431–3.201)	** < 0.001**	2.748 (1.747–4.323)	** < 0.001**
Adverse	141	3.988 (2.762–5.758)	** < 0.001**	3.994 (2.426–6.575)	** < 0.001**
Allo-HSCT	502		** < 0.001**		
No	308	Reference		Reference	
Yes	194	0.260 (0.176–0.384)	** < 0.001**	0.162 (0.105–0.250)	** < 0.001**
DNMT3A mutation	502		0.403		
WT	393	Reference			
MUT	109	1.160 (0.823–1.634)	0.397		
TET2 mutation	502		**0.006**		
WT	426	Reference		Reference	
MUT	76	1.735 (1.195–2.517)	**0.004**	1.649 (1.131–2.405)	**0.009**
IDH1 mutation	502		0.228		
WT	470	Reference			
MUT	32	1.407 (0.828–2.392)	0.207		
IDH2 mutation	502		0.701		
WT	449	Reference			
MUT	53	1.095 (0.693–1.729)	0.698		
WT1 mutation	502		0.576		
WT	431	Reference			
MUT	71	1.126 (0.747–1.699)	0.571		
Bone marrow blasts percentage, median (IQR)	493	1.001 (0.994–1.008)	0.726		
WBC(× 10^9^/L), median (IQR)	451	1.001 (0.998–1.004)	0.466		
Hemoglobin(g/L), median (IQR)	449	1.002 (0.996–1.008)	0.501		
Platelets(× 10^9^/L), median (IQR)	449	1.000 (0.998–1.003)	0.666		
Age	500	1.023 (1.013–1.033)	** < 0.001**	1.002 (0.992–1.013)	0.714

*P* values less than 0.05 are highlighted in bold

*Allo-HSCT* allogenic hematopoietic stem cell transplantation, *FAB* French–American–British classification, *WBC* white blood cell, *DNMT3A* DNA methyltransferase 3 alpha; *IDH1* isocitrate dehydrogenase 1, *IDH2* isocitrate dehydrogenase 2, *TET2* tet methylcytosine dioxygenase 2, *WT1* Wilms tumor 1

The prognostic value of TET2 gene mutations in AML patients with various risk statuses based on the 2022 ELN risk classification was further analyzed. Notably, there is still a lack of valid prognostic indicators for patients at intermediate risk. Among the 130 patients in the Peking cohort who were at intermediate risk, those with TET2 mutations presented lower OS compared to those with TET2 WT (HR = 1.967, *p* = 0.05) (Supplementary Table S4). We also performed subgroup analysis based on different mutation profiles. We found that the presence of TET2 mutations significantly worsened the OS of NPM1+ AML patients (*p* = 0.005), with no significant impact observed for the other AML subgroups with FLT3-ITD, DNMT3A, ASXL1, U2AF1, or BCOR mutations (Supplementary Figure S2). The impact of TET2 mutations on response to induction chemotherapy and EFS in all the AML patients in the Peking cohort was also investigated. The relevant results can be found in Supplementary Tables S5 and S6. According to univariate Cox regression analysis for EFS, TET2 mutations were identified as risk factor (hazard ratio (HR) = 1.509, *p* = 0.013).

### Survival outcomes

**Fig. 3:**
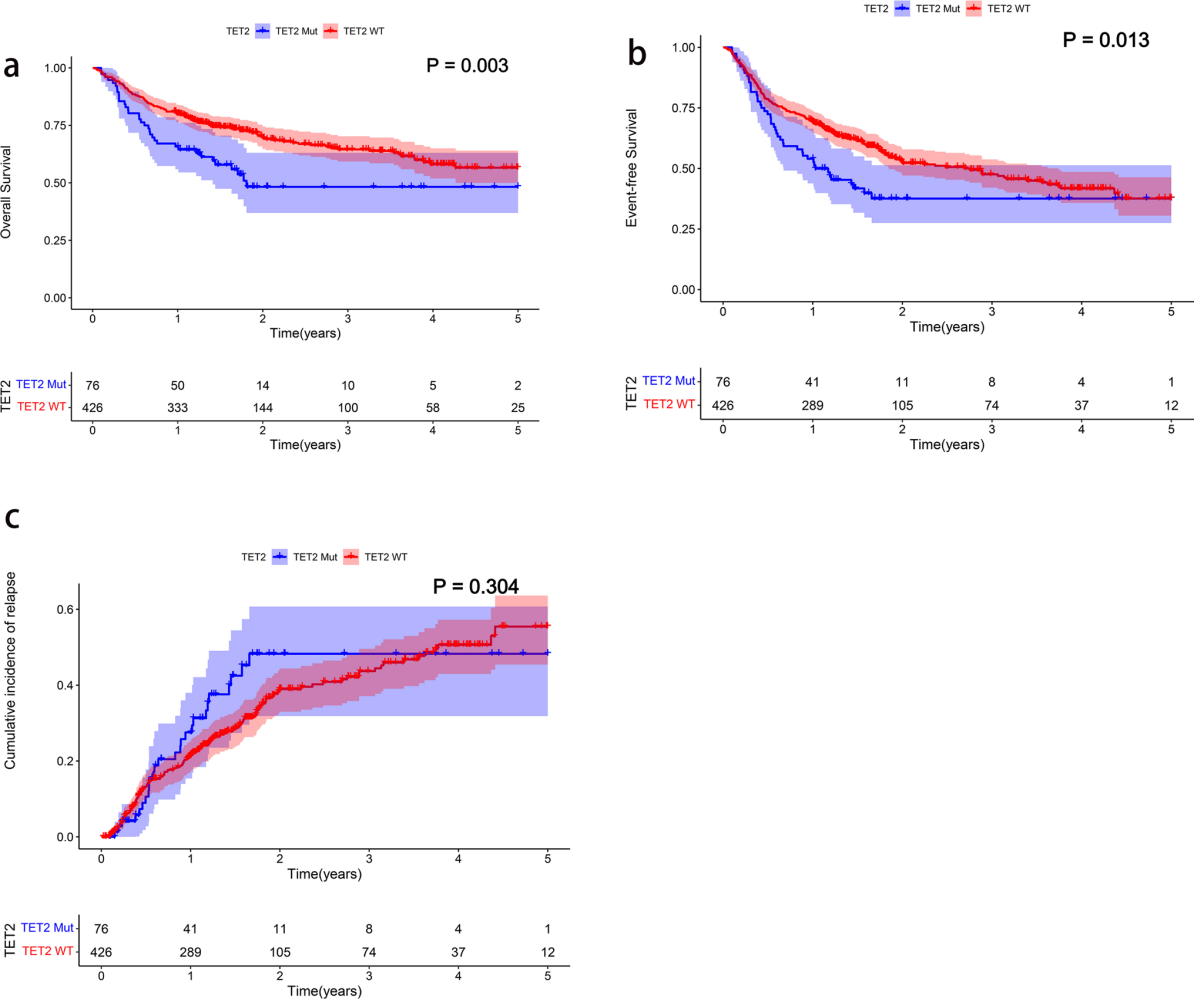
The prognostic significance of TET2 mutation status was evaluated in 502 patients in the Peking cohort. **a** Kaplan–Meier estimates of OS. **b** Kaplan–Meier estimates of EFS. **c** Cumulative incidence of relapse

Data from the Beat AML database were divided into TET2-mutant and TET2-WT groups, and the 3-year OS was lower in the former group (15.7% vs. 25.1%, *p* = 0.011) (Supplementary Figure S3). Retrospective analysis of AML patients from the Peking cohort also revealed shorter 3-year OS (48.2% vs. 64.6%, *p* = 0.003) and 3-year EFS (37.6% vs. 47.6%, *p* = 0.013) in the TET2-mutated group than in the non-TET2-mutated group (Fig. 3a, b). However, there was no statistically significant difference in CIR between the two groups. (*p* = 0.304) (Fig. 3c).

### Prognostic analysis of patients with TET2 mutations

We constructed a prognostic survival nomogram based on univariate and multivariate analyses of OS in 76 AML patients harboring TET2 mutations (Table S7). Three predictors (WT1 expression level at diagnosis, complete remission (CR) in phase 1 chemotherapy, and HSCT) were ultimately incorporated into the model. To predict the individual probabilities of 2-, 3-, and 4-year OS, the points corresponding to the three variables were combined. The predictive accuracy for OS, evaluated using the C-index, was 0.75 (95% CI = 0.709–0.791) (Supplementary Figure S4).

### Identification of DEGs and enrichment analyses

**Fig. 4:**
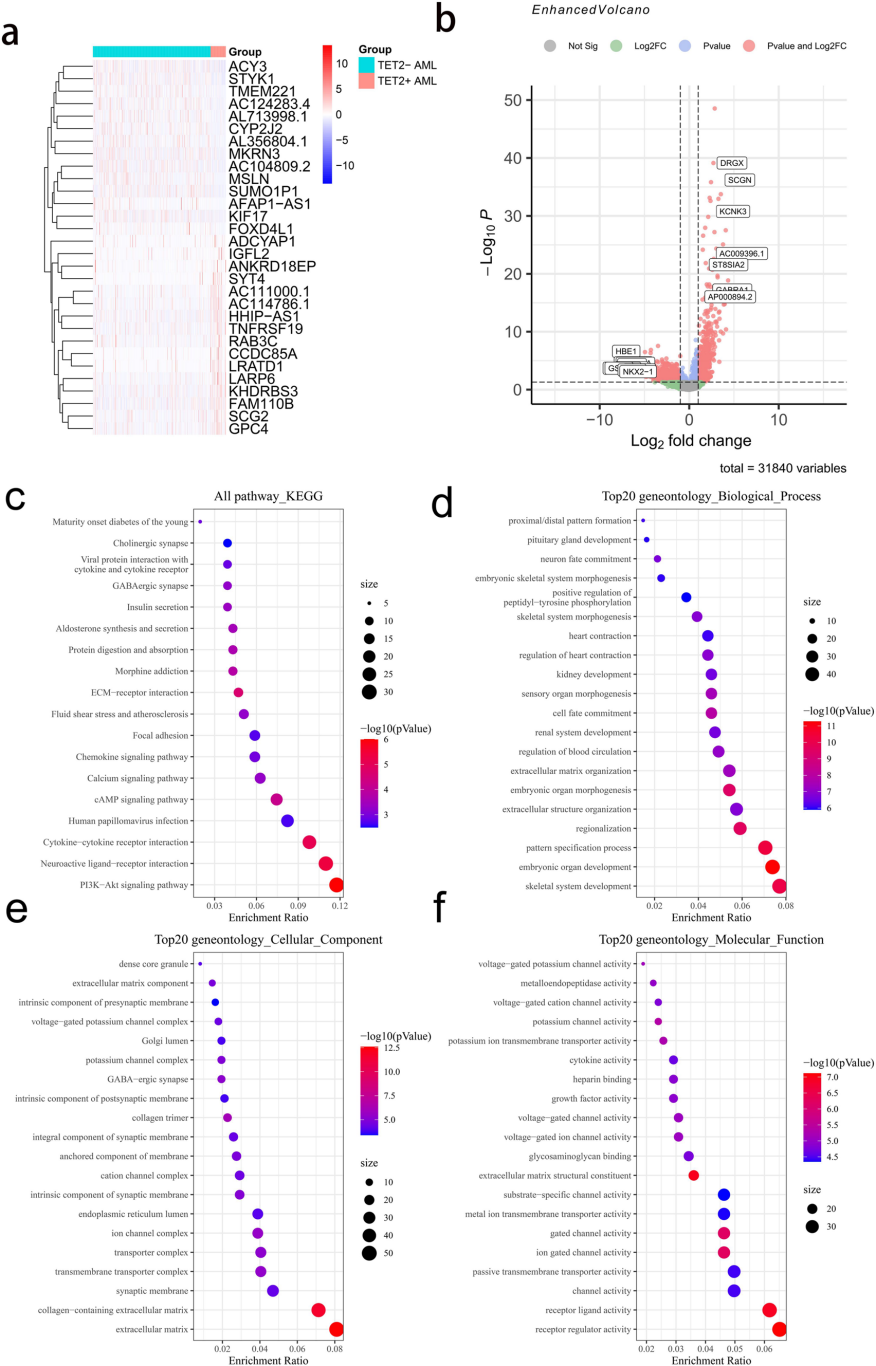
DAVID enrichment analysis of DEGs. **a** Heatmap of DEGs between TET2-Mut and TET2-WT AML patients. **b** Volcano plot for DEGs. **c** KEGG pathway analysis of DEGs. **d**–**f** GO enrichment analysis of DEGs

The aforementioned results suggest that TET2 mutations adversely affect the prognosis of AML patients. To further investigate the genes and signaling pathways associated with TET2 mutations, we identified TET2 mutation-associated DEGs through screening of RNA-Seq data for 50 patients with TET2 mutations and patients with the WT TET2 gene in the Beat AML database. Using a fold change (FC)|≥ 1.0 and FDR < 0.05 as selection criteria, a total of 1042 DEGs, including 507 upregulated and 535 downregulated genes, were identified. An expression heatmap and a volcano plot of the detected DEGs are shown in Fig. 4a and b, respectively. Gene ontology (GO) enrichment and Kyoto Encyclopedia of Genes and Genomes (KEGG) pathway analyses highlighted significant enrichment of these DEGs in categories associated with the PI3K-Akt signaling pathway (Fig. 4c) and in multiple processes potentially influencing cancer development (Fig. 4d–f). Identical differential gene and KEGG enrichment analyses were performed using the GSE32246 dataset, revealing that 783 DEGs were also predominantly enriched in the PI3K-Akt signaling pathway (Figure S6a, b). The genes involved in the PI3K-Akt signaling pathway include COMP/CREB3L3/GNG8/CREB3L1, among others. GSEA revealed significant activation of the chemokine signaling pathway and calcium signaling pathway in TET2+ AML patients (both *p* < 0.01, NES = 1.593 and NSE = 1.455; Supplementary Figure S5a). We also utilized the Beat AML database to analyze DEGs between TET2+ and TET2− AML patients and healthy controls. Our differential gene expression analysis produced a Venn diagram revealing 3710 genes that overlapped between the two groups (Supplementary Figure S5b).

### PPI network and module screening

**Fig. 5:**
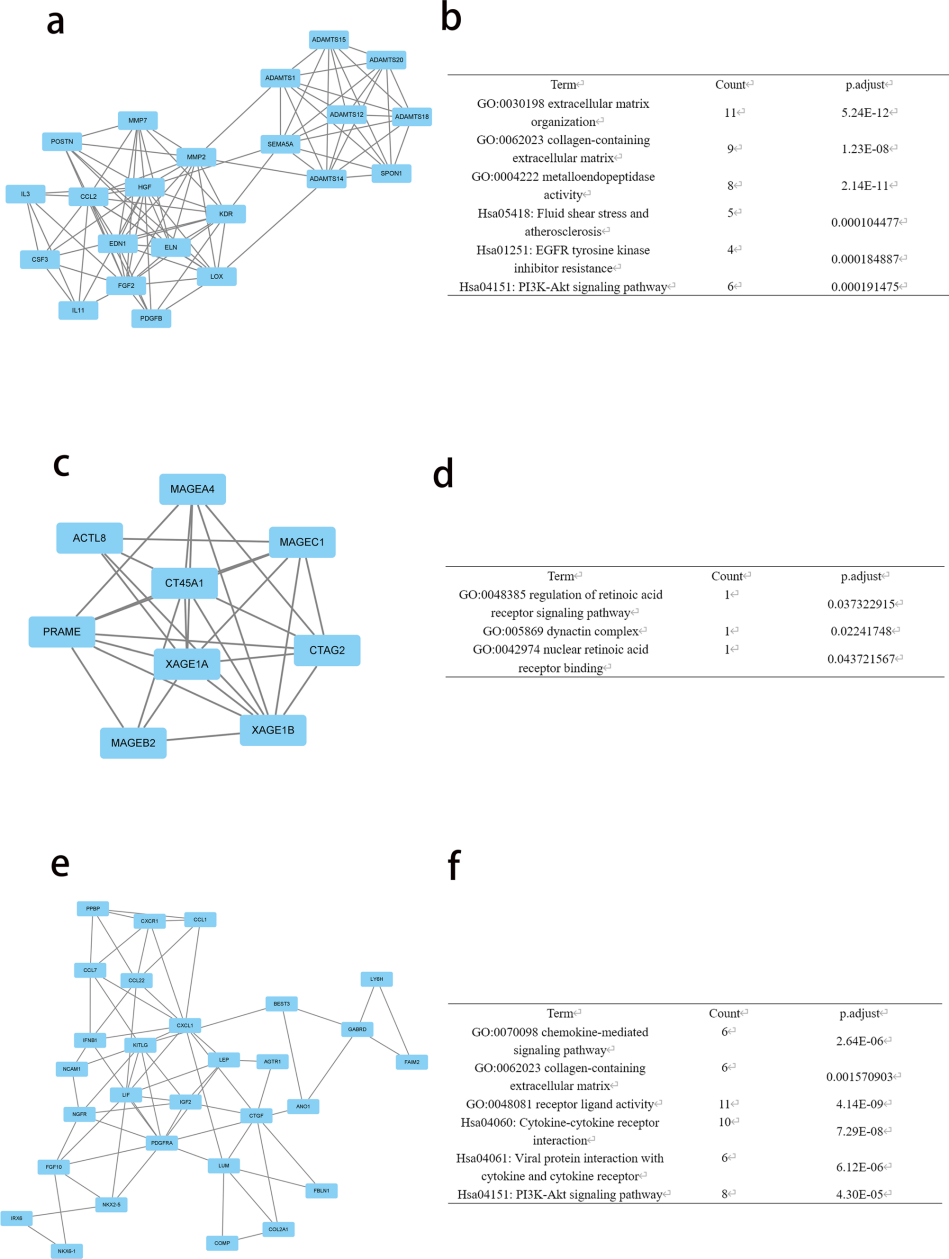
The three most significant modules were obtained from the PPI network. **a**, **b** Module 1’s PPI network and corresponding GO and KEGG analyses (22 hub genes). **c**, **d** Module 2’s PPI network and corresponding GO and KEGG analyses (9 hub genes). **e**, **f** Module 3’s PPI network and corresponding GO and KEGG analyses (29 hub genes)

We further screened the STRING database to investigate interactions between hub genes and the DEGs identified above. The top 10 hub genes, as ranked by degree, were BMP4, FGF2, CCL2, HGF, CTGF, EDN1, FOXA2, CXCL1, FGF10, and GABRA1. Among the hub genes, BMP4 had the highest node degree (48). We then discerned the gene modules in the PPI network using the MCODE plugin in Cytoscape. The top three significant modules were subsequently selected and subjected to GO and KEGG pathway enrichment analyses (Fig. 5). The results showed that the genes in modules 1–3 are mainly associated with the PI3K-Akt signaling pathway, regulation of the retinoic acid receptor signaling pathway, the chemokine-mediated signaling pathway, extracellular matrix organization, the dynactin complex, and receptor ligand activity.

## Discussion

The TET protein family consists of TET1, TET2, and TET3, which are key enzymes involved in the regulation of DNA demethylation. Among the TET family members, only TET2 is mutated in hematologic malignancies [23]. Identification of recurrent mutations in TET2 in hematological malignancies suggests that epigenetic regulation is essential for hematopoietic cell transformation [24]. However, the role of TET2 in leukemogenesis is currently unclear. A few studies have addressed its clinical significance in AML, but with inconsistent and even controversial results [14, 25–27].

Our study is the first to combine a large clinical cohort with data from a public AML database to evaluate the clinical significance and potential molecular mechanisms of TET2 mutations. Similar to the results of previous studies [26] and reaffirming the notion that TET2 mutations are associated with age-related clonal hematopoiesis, we found that the median age of AML patients with TET2 mutations was significantly greater than that of patients who did not carry such mutations. Both TET2 and DNMT3A are important epigenetic regulators, and previous studies have shown a greater frequency of DNMT3A mutations in patients with cytogenetically normal (CN) AML harboring TET2 mutations [27]. However, our study indicated no significant concomitant relationship between TET2 and DNMT3A mutation status in the entire AML cohort.

Among our cohort of 76 AML patients carrying TET2 mutations, we found no significant effect of TET2 mutation number or frequency on OS by Cox regression analysis. Intriguingly, we nevertheless observed that three OS indicators derived from regression analysis (WT1 expression level at diagnosis, CR in phase 1 chemotherapy, and HSCT) affected the prognosis of patients with TET2-mutated AML. Previous studies have shown that TET2 mutations may lead to abnormal DNA repair activity and that TET2 interacts with WT1 to activate gene expression. Indeed, defective DNA repair has been reported upon disrupted TET2-WT1 binding [28]. We hypothesized that WT1 overexpression may improve the DNA repair capacity of TET2+ AML patients, which may be the mechanism leading to poor prognosis in patients with TET2 mutations.

Multivariate analysis of 502 AML patients treated at our institution revealed that TET2 mutations independently reduced overall and event-free survival and were also risk factors for relapse. In turn, analysis of the Beat AML database indicated that TET2 mutations contribute to worse OS. We evaluated the TET2 mutation status and the three clinical indicators mentioned above and constructed a clinical predictive nomogram that accurately identified patients with more favorable prognosis. This novel model may help physicians to intervene earlier to improve patient prognosis and reduce patient mortality.

The newest 2022 ELN AML guidelines have recently been published; however, in this risk classification system, the prognostic significance of TET2 mutations in different risk groups has not been determined, and robust prognostic indicators are still lacking for patients in the intermediate-risk group. Interestingly, our study suggested that TET2 mutations are significant predictors of OS in the intermediate-risk group of AML patients.

To assess the potential mechanisms by which TET2 mutations adversely influence AML prognosis, we analyzed the RNA-Seq dataset downloaded from the Beat AML cohort to identify key pathways and genes associated with TET2 mutations. GO and KEGG enrichment analyses of the 1042 DEGs identified upon comparison of patients carrying a TET2-mutated or nonmutated gene indicated that the DEGs in AML patients with TET2 mutations were enriched mainly in the PI3K-AKT signaling pathway, skeletal system development, the extracellular matrix, and receptor regulator activity. Recent preclinical and clinical studies have elucidated the significance of PI3K-AKT signaling in AML by revealing that the activation state of the PI3K-Akt-mTOR axis may be a reflection of clonal heterogeneity and differential gene expression dynamics. The PI3K-Akt-mTOR pathway is critical for disease progression and chemotherapeutic sensitivity in multiple cancers, and overactivation of this pathway has an adverse prognostic impact on acute myeloid leukemia (AML) [29, 30]. Taking together these findings and our evidence that TET2 mutations are associated with poor prognosis in AML patients, we speculate that activation of the PI3K-AKT pathway might at least in part mediate the negative effect of TET2 mutations on AML prognosis. Although PI3K-AKT pathway inhibitors have shown limited efficacy in multiple clinical trials, they may yield better results when used in combination with other agents, such as USP15, a TET2 deubiquitinase and inhibitor [31, 32]. Lei-lei Chen et al. demonstrated that USP15 is a deubiquitinase and inhibitor of TET2. KEGG enrichment analysis of 181 IFN-γ-induced genes oppositely regulated by Usp15 and Tet2 KO revealed that the differentially expressed genes were enriched in the chemokine signaling pathway [31]. These findings are consistent with our KEGG enrichment analysis. Previous investigations have demonstrated the presence of the CCL2 protein as a cytokine within the IL-17 signaling pathway that participates in autoimmune disorders, inflammatory processes, and the tumor microenvironment. In the context of AML, expression of IL-17 has been linked to unfavorable prognosis. Elevated levels of CCL2 can facilitate monocyte migration, intensify inflammation, and stimulate the production of proangiogenic factors. Notably, CCL2 overexpression was also observed in AML patients [33, 34]. Our investigation revealed greater expression of CCL2 in TET2 + AML patients than in TET2-AML patients (|log FC|= 4.2, FDR < 0.001). Consequently, targeting CCL2 may be a promising therapeutic approach for AML patients harboring TET2 mutations.

A limitation of our study is that we investigated the impact of TET2 mutations on AML prognosis and disease progression but did not specifically address the underlying molecular mechanisms involved. Thus, molecular studies are needed to clarify the roles of different TET2 mutations in AML. Another limitation of our study is that certain clinical information, including the initial white blood cell count, platelet count, hemoglobin level, and bone marrow blast cell proportion, was missing for the Peking AML cohort. These missing values were inadvertently omitted due to constraints in data collection and availability.

## Conclusions

Our study scrutinized the prognostic implications of TET2 mutations in an AML cohort from our institution and validated the results in the Beat AML patient population. Notably, the prognostic utility of TET2 mutational status was specifically assessed within the cohort of intermediate-risk AML patients, a subgroup currently without reliable prognostic markers. Moreover, we identified several genes and signaling pathways that are associated with TET2 mutations in AML, suggesting that, subject to experimental validation, these genes and signaling pathways may be used to develop efficacious targeted therapeutic strategies.

## Supplementary Information

Below is the link to the electronic supplementary material.

Supplementary file1 (XLSX 239 kb)

Supplementary file2 (DOCX 13284 kb)

## Data Availability

The datasets generated and/or analyzed during the present study are available from the corresponding author upon reasonable request.
