# Supplementary material for: TET2 mutations contribute to adverse prognosis in acute myeloid leukemia (AML): results from a comprehensive analysis of 502 AML cases and the Beat AML public database
Source: Clin Exp Med. 2024 Feb 13;24(1):35. doi: 10.1007/s10238-024-01297-0 (PMC10864580; doi:10.1007/s10238-024-01297-0)
Supplement: Supplementary file 2 — Supplementary file2 (DOCX 13284 kb) [file 10238_2024_1297_MOESM2_ESM.docx]

**Supplementary Materials**

**Table S1.** Patient characteristics of the Peking cohort categorized by TET2 mutation status.

| Characteristic | Category | | | TET2 wild type | | TET2 mutation | | P value |
| --- | --- | --- | --- | --- | --- | --- | --- | --- |
| n |  | | | 426 | | 76 | |  |
| NPM1 mutations, n (%) | WT | | | 321 (75.4) | | 54 (71.1) | | 0.515 |
|  | MUT | | | 105 (24.6) | | 22 (28.9) | |  |
| FLT3-ITD mutations, n (%) | WT | | | 345 (81.0) | | 61 (80.3) | | 1.000 |
|  | MUT | | | 81 (19.0) | | 15 (19.7) | |  |
| FLT3-TKD mutations, n (%) | WT | | | 394 (92.5) | | 71 (93.4) | | 0.961 |
|  | MUT | | | 32 (7.5) | | 5 (6.6) | |  |
| DNMT3A mutations, n (%) | WT | | | 336 (78.9) | | 57 (75.0) | | 0.546 |
|  | MUT | | | 90 (21.1) | | 19 (25.0) | |  |
| TP53 mutations, n (%) | WT | | | 397 (93.2) | | 69 (90.8) | | 0.612 |
|  | MUT | | | 29 (6.8) | | 7 (9.2) | |  |
| RUNX1 mutations, n (%) | WT | | | 384 (90.1) | | 67 (88.2) | | 0.748 |
|  | MUT | | | 42 (9.9) | | 9 (11.8) | |  |
| ASXL1 mutations, n (%) | WT | | | 384 (90.1) | | 60 (78.9) | | 0.009 |
|  | MUT | | | 42 (9.9) | | 16 (21.1) | |  |
| WT1 mutations, n (%) | | WT | 365 (85.7) | | 66 (86.8) | | 0.929 | |
|  | | MUT | 61 (14.3) | | 10 (13.2) | |  | |
| KIT mutations, n (%) | WT | | | 376 (88.3) | | 70 (92.1) | | 0.434 |
|  | MUT | | | 50 (11.7) | | 6 (7.9) | |  |
| SF3B1mutations, n (%) | WT | | | 416 (97.7) | | 76 (100) | | 0.372 |
|  | MUT | | | 10 (2.3) | | 0 (0.0) | |  |
| U2AF1 mutations. n (%) | WT | | | 403 (94.6) | | 68 (89.5) | | 0.115 |
|  | MUT | | | 23 (5.4) | | 8 (10.5) | |  |
| SRSF2 mutations, n (%) | WT | | | 414 (97.2) | | 69 (90.8) | | 0.015 |
|  | MUT | | | 12 (2.8) | | 7 (9.2) | |  |
| EZH2 mutations, n (%) | WT | | | 409 (96.0) | | 71 (93.4) | | 0.356 |
|  | MUT | | | 17 (4.0) | | 5 (6.6) | |  |
| STAG2 mutation, n (%) | WT | | | 406 (95.3) | | 70 (92.1) | | 0.259 |
|  | MUT | | | 20 (4.7) | | 6 (7.9) | |  |
| GATA2 mutations, n (%) | WT | | | 398 (93.4) | | 75 (98.7) | | 0.104 |
|  | MUT | | | 28 (6.6) | | 1 (1.3) | |  |
| IDH1 mutations, n (%) | WT | | | 398 (93.4) | | 72 (94.7) | | 0.803 |
|  | MUT | | | 28 (6.6) | | 4 (5.3) | |  |
| IDH2 mutations, n (%) | WT | | | 377 (88.5) | | 72 (94.7) | | 0.153 |
|  | MUT | | | 49 (11.5) | | 4 (5.3) | |  |
| SMC3 mutations, n (%) | WT | | | 419 (98.4) | | 72 (94.7) | | 0.069 |
|  | MUT | | | 7 (1.6) | | 4 (5.3) | |  |
| BCOR mutations, n (%) | WT | | | 385 (90.4) | | 66 (86.8) | | 0.463 |
|  | MUT | | | 41 (9.6) | | 10 (13.2) | |  |
| ZRSR2 mutations, n (%) | WT | | | 419 (98.4) | | 74 (97.4) | | 0.631 |
|  | MUT | | | 7 (1.6) | | 2 (2.6) | |  |

NPM1, Nucleophosmin1; FLT3-ITD, FMS-like tyrosine kinase 3-Internal tandem duplication.

**Table S2.** AML-related mutation Characteristics in Peking Cohort Categorized by TET2 Mutation Frequency

| Characteristics | TET2 mutation frequency_ High | TET2 mutation frequency_ Low | P value |
| --- | --- | --- | --- |
| n | 40 | 36 |  |
| NPM1 mutations, n (%) |  |  | 0.831 |
| WT | 28 (36.8%) | 26 (34.2%) |  |
| MUT | 12 (15.8%) | 10 (13.2%) |  |
| FLT3-ITD mutations, n (%) |  |  | 0.523 |
| WT | 31 (40.8%) | 30 (39.5%) |  |
| MUT | 9 (11.8%) | 6 (7.9%) |  |
| FLT3-TKD mutations, n (%) |  |  | 1.000 |
| WT | 37 (48.7%) | 34 (44.7%) |  |
| MUT | 3 (3.9%) | 2 (2.6%) |  |
| DNMT3A mutations, n (%) |  |  | 0.596 |
| WT | 29 (38.2%) | 28 (36.8%) |  |
| MUT | 11 (14.5%) | 8 (10.5%) |  |
| TP53 mutations, n (%) |  |  | 1.000 |
| MUT | 4 (5.3%) | 3 (3.9%) |  |
| WT | 36 (47.4%) | 33 (43.4%) |  |
| RUNX1 mutations, n (%) |  |  | 1.000 |
| WT | 35 (46.1%) | 32 (42.1%) |  |
| MUT | 5 (6.6%) | 4 (5.3%) |  |
| ASXL1 mutations, n (%) |  |  | 0.374 |
| MUT | 10 (13.2%) | 6 (7.9%) |  |
| WT | 30 (39.5%) | 30 (39.5%) |  |
| WT1 mutations, n (%) |  |  | 1.000 |
| WT | 35 (46.1%) | 31 (40.8%) |  |
| MUT | 5 (6.6%) | 5 (6.6%) |  |
| KIT mutations, n (%) |  |  | 0.158 |
| WT | 39 (51.3%) | 31 (40.8%) |  |
| MUT | 1 (1.3%) | 5 (6.6%) |  |
| U2AF1, n (%) |  |  | 0.595 |
| WT | 37 (48.7%) | 31 (40.8%) |  |
| MUT | 3 (3.9%) | 5 (6.6%) |  |
| SRSF2 mutations, n (%) |  |  | 0.517 |
| WT | 35 (46.1%) | 34 (44.7%) |  |
| MUT | 5 (6.6%) | 2 (2.6%) |  |
| EZH2 mutations, n (%) |  |  | 0.421 |
| WT | 36 (47.4%) | 35 (46.1%) |  |
| MUT | 4 (5.3%) | 1 (1.3%) |  |
| STAG2 mutations, n (%) |  |  | 1.000 |
| WT | 37 (48.7%) | 33 (43.4%) |  |
| MUT | 3 (3.9%) | 3 (3.9%) |  |
| GATA2 mutations, n (%) |  |  | 0.474 |
| WT | 40 (52.6%) | 35 (46.1%) |  |
| MUT | 0 (0%) | 1 (1.3%) |  |
| IDH1 mutations, n (%) |  |  | 1.000 |
| WT | 38 (50%) | 34 (44.7%) |  |
| MUT | 2 (2.6%) | 2 (2.6%) |  |
| IDH2 mutations, n (%) |  |  | 0.533 |
| WT | 39 (51.3%) | 33 (43.4%) |  |
| MUT | 1 (1.3%) | 3 (3.9%) |  |
| SMC3 mutations, n (%) |  |  | 0.533 |
| WT | 39 (51.3%) | 33 (43.4%) |  |
| MUT | 1 (1.3%) | 3 (3.9%) |  |
| BCOR mutation, n (%) |  |  | 1.000 |
| WT | 35 (46.1%) | 31 (40.8%) |  |
| MUT | 5 (6.6%) | 5 (6.6%) |  |
| ZRSR2 mutations, n (%) |  |  | 1.000 |
| WT | 39 (51.3%) | 35 (46.1%) |  |
| MUT | 1 (1.3%) | 1 (1.3%) |  |

NPM1, Nucleophosmin1; FLT3-ITD, FMS-like tyrosine kinase 3-Internal tandem duplication.

**Table S3.** Univariate and multivariate analysesof OS in AML patients from Beat AML database.

| Characteristics | Total(N) | Univariate analysis | |  | Multivariate analysis | |
| --- | --- | --- | --- | --- | --- | --- |
|  |  | Hazard ratio (95% CI) | P value |  | Hazard ratio (95% CI) | P value |
| **Allo-HSCT** | 604 |  | 0.403 |  |  |  |
| No | 581 | Reference |  |  |  |  |
| Yes | 23 | 0.823 (0.516 - 1.315) | 0.416 |  |  |  |
| **CEBPA double mutation** | 604 |  | **0.003** |  |  |  |
| WT | 593 | Reference |  |  | Reference |  |
| MUT | 11 | 0.131 (0.018 - 0.933) | **0.042** |  | 0.134 (0.018 - 0.965) | **0.046** |
| **ELN2017** | 604 |  | **< 0.001** |  |  |  |
| Favorable | 175 | Reference |  |  | Reference |  |
| Intermediate | 211 | 1.515 (1.123 - 2.045) | **0.007** |  | 1.273 (0.938 - 1.726) | 0.121 |
| Adverse | 218 | 2.245 (1.677 - 3.004) | **< 0.001** |  | 1.559 (1.131 - 2.150) | **0.007** |
| **FLT3-ITD mutation** | 604 |  | **0.039** |  |  |  |
| WT | 456 | Reference |  |  | Reference |  |
| MUT | 148 | 1.280 (1.017 - 1.612) | **0.036** |  | 1.352 (1.065 - 1.716) | **0.013** |
| **Gender** | 604 |  | **0.014** |  |  |  |
| Female | 270 | Reference |  |  | Reference |  |
| Male | 334 | 1.306 (1.055 - 1.616) | **0.014** |  | 1.310 (1.053 - 1.629) | **0.015** |
| **NPM1 mutations** | 602 |  | 0.887 |  |  |  |
| WT | 443 | Reference |  |  |  |  |
| MUT | 159 | 1.017 (0.802 - 1.291) | 0.887 |  |  |  |
| **TET2 mutations** | 604 |  | **0.010** |  |  |  |
| WT | 535 | Reference |  |  | Reference |  |
| MUT | 69 | 1.533 (1.127 - 2.087) | **0.007** |  | 1.801 (1.316 - 2.466) | **< 0.001** |
| **ASXL1 mutations** | 604 |  | 0.062 |  |  |  |
| WT | 558 | Reference |  |  | Reference |  |
| MUT | 46 | 1.439 (0.999 - 2.072) | 0.050 |  | 1.247 (0.855 - 1.820) | 0.252 |
| **DNMT3A mutations** | 604 |  | 0.908 |  |  |  |
| WT | 479 | Reference |  |  |  |  |
| MUT | 125 | 1.016 (0.782 - 1.319) | 0.908 |  |  |  |
| **RUNX1 mutations** | 604 |  | 0.069 |  |  |  |
| WT | 538 | Reference |  |  | Reference |  |
| MUT | 66 | 1.353 (0.988 - 1.853) | 0.059 |  | 1.155 (0.823 - 1.622) | 0.404 |
| **TP53 mutations** | 604 |  | **< 0.001** |  |  |  |
| WT | 556 | Reference |  |  | Reference |  |
| MUT | 48 | 4.044 (2.919 - 5.603) | **< 0.001** |  | 4.020 (2.782 - 5.809) | **< 0.001** |
| **IDH1 mutations** | 604 |  | 0.317 |  |  |  |
| WT | 555 | Reference |  |  |  |  |
| MUT | 49 | 0.822 (0.554 - 1.220) | 0.330 |  |  |  |
| **IDH2 mutations** | 604 |  | 0.606 |  |  |  |
| WT | 534 | Reference |  |  |  |  |
| MUT | 70 | 0.921 (0.673 - 1.261) | 0.609 |  |  |  |

HSCT, hematopoietic stem cell transplantation; ELN2017, 2017 European LeukemiaNet (ELN) classification guideline.

**Table S4.** Univariate and multivariate analysesof OS in 2022 ELN intermediate-risk AML patients from Peking cohort.

| Characteristics | Total(N) | Univariate analysis | |  | Multivariate analysis | |
| --- | --- | --- | --- | --- | --- | --- |
|  |  | Hazard ratio (95% CI) | P value |  | Hazard ratio (95% CI) | P value |
| **Gender** | 130 |  | 0.223 |  |  |  |
| Female | 72 | Reference |  |  |  |  |
| Male | 58 | 1.420 (0.809 - 2.494) | 0.222 |  |  |  |
| **FAB type** | 130 |  | 0.685 |  |  |  |
| M0 | 2 | Reference |  |  |  |  |
| M1 | 3 | 1.261 (0.078 - 20.429) | 0.870 |  |  |  |
| M2 | 77 | 0.857 (0.116 - 6.334) | 0.880 |  |  |  |
| M4 | 31 | 1.284 (0.168 - 9.808) | 0.810 |  |  |  |
| M5 | 16 | 0.964 (0.116 - 8.033) | 0.973 |  |  |  |
| M7 | 1 | 5.008 (0.306 - 81.947) | 0.259 |  |  |  |
| **Cytogenetic risk stratification** | 130 |  | 0.197 |  |  |  |
| Intermediate | 128 | Reference |  |  |  |  |
| Adverse | 2 | 0.000 (0.000 - Inf) | 0.996 |  |  |  |
| **Allo-HSCT** | 130 |  | **< 0.001** |  |  |  |
| No | 72 | Reference |  |  | Reference |  |
| Yes | 58 | 0.266 (0.138 - 0.513) | **< 0.001** |  | 0.305 (0.154 - 0.606) | **< 0.001** |
| **DNMT3A mutations** | 130 |  | 0.051 |  |  |  |
| WT | 80 | Reference |  |  | Reference |  |
| MUT | 50 | 1.754 (1.001 - 3.074) | 0.050 |  | 1.289 (0.697 - 2.383) | 0.418 |
| **TET2 mutations** | 130 |  | **0.041** |  |  |  |
| WT | 107 | Reference |  |  | Reference |  |
| MUT | 23 | 2.077 (1.079 - 3.997) | **0.029** |  | 1.967 (1.001 - 3.865) | 0.050 |
| **IDH1 mutations** | 130 |  | **0.015** |  |  |  |
| WT | 124 | Reference |  |  | Reference |  |
| MUT | 6 | 3.974 (1.547 - 10.209) | **0.004** |  | 2.601 (0.957 - 7.067) | 0.061 |
| **IDH2 mutations** | 130 |  | 0.450 |  |  |  |
| WT | 109 | Reference |  |  |  |  |
| MUT | 21 | 0.741 (0.332 - 1.657) | 0.466 |  |  |  |
| **WT1 mutations** | 130 |  | 0.669 |  |  |  |
| WT | 107 | Reference |  |  |  |  |
| MUT | 23 | 1.161 (0.591 - 2.279) | 0.665 |  |  |  |
| Bone marrow blasts percentage, median (IQR) | 126 | 1.000 (0.987 - 1.013) | 0.966 |  |  |  |
| WBC(×10^9^/L) , median (IQR) | 119 | 0.999 (0.994 - 1.004) | 0.782 |  |  |  |
| Hemoglobin(g/L), median (IQR) | 118 | 1.005 (0.993 - 1.016) | 0.409 |  |  |  |
| Platelets(×10^9^/L) , median (IQR) | 118 | 1.000 (0.997 - 1.004) | 0.825 |  |  |  |
| age | 129 | 1.008 (0.990 - 1.027) | 0.385 |  |  |  |

Allo-HSCT, allogenic hematopoietic stem cell transplantation; FAB, French–American–British classification; WBC, white blood cells; DNMT3A, DNA methyltransferase 3 alpha; IDH1, isocitrate dehydrogenase 1; IDH2, isocitrate dehydrogenase 2; TET2, tet methylcytosine dioxygenase 2; WT1, Wilms tumor 1.

**Table S5.** Univariate and multivariate analyses of CR1 in entire AML patients from Peking cohort.

| Characteristics | Total(N) | Univariate analysis | |  | Multivariate analysis | |
| --- | --- | --- | --- | --- | --- | --- |
|  |  | Odds Ratio (95% CI) | P value |  | Odds Ratio (95% CI) | P value |
| age | 500 | 1.014 (1.002 - 1.026) | **0.024** |  | 1.014 (1.000 - 1.029) | 0.050 |
| Gender | 502 |  |  |  |  |  |
| male | 262 | Reference |  |  |  |  |
| female | 240 | 0.992 (0.693 - 1.421) | 0.967 |  |  |  |
| FAB type | 502 | 0.998 (0.859 - 1.160) | 0.980 |  |  |  |
| Cytogenetic risk stratification | 502 |  |  |  |  |  |
| Intermediate | 336 | Reference |  |  | Reference |  |
| favorable | 91 | 0.383 (0.216 - 0.678) | **< 0.001** |  | 0.759 (0.368 - 1.566) | 0.455 |
| Adverse | 75 | 3.768 (2.200 - 6.455) | **< 0.001** |  | 1.144 (0.532 - 2.461) | 0.730 |
| 2022 ELN risk classification | 502 |  |  |  |  |  |
| favorable | 231 | Reference |  |  | Reference |  |
| Intermediate | 130 | 2.168 (1.359 - 3.458) | **0.001** |  | 2.312 (1.288 - 4.149) | **0.005** |
| Adverse | 141 | 6.112 (3.852 - 9.698) | **< 0.001** |  | 5.840 (2.830 - 12.051) | **< 0.001** |
| Bone marrow blasts percentage, median (IQR) | 493 | 1.005 (0.997 - 1.013) | 0.255 |  |  |  |
| WBC(×10^9^/L) , median (IQR) | 451 | 1.001 (0.998 - 1.005) | 0.501 |  |  |  |
| Hemoglobin(g/L), median (IQR) | 449 | 0.996 (0.989 - 1.004) | 0.326 |  |  |  |
| Platelets(×10^9^/L) , median (IQR) | 449 | 1.001 (0.998 - 1.003) | 0.504 |  |  |  |
| **DNMT3A mutations** | 502 |  |  |  |  |  |
| Yes | 109 | Reference |  |  |  |  |
| No | 393 | 1.308 (0.839 - 2.041) | 0.236 |  |  |  |
| **TET2 mutations** | 502 |  |  |  |  |  |
| No | 426 | Reference |  |  |  |  |
| Yes | 76 | 1.419 (0.868 - 2.321) |  |  |  |  |
| **WT1 mutations** | 502 |  |  |  |  |  |
| No | 431 | Reference |  |  | Reference |  |
| Yes | 71 | 0.834 (0.494 - 1.409) | 0.498 |  | 0.756 (0.402 - 1.420) | 0.384 |

CR1, complete remission in phase 1 chemotherapy; ELN2017, 2017 European LeukemiaNet (ELN) classification guideline.

**Table S6.** Univariate and multivariate analyses of EFS in entire AML patients from Peking cohort.

| Characteristics | Total(N) | Univariate analysis | |  | Multivariate analysis | |
| --- | --- | --- | --- | --- | --- | --- |
|  |  | Hazard ratio (95% CI) | P value |  | Hazard ratio (95% CI) | P value |
| **Gender** | 502 |  | **0.037** |  |  |  |
| Female | 240 | Reference |  |  | Reference |  |
| Male | 262 | 1.307 (1.016 - 1.683) | **0.038** |  | 1.282 (0.977 - 1.682) | 0.073 |
| **FAB type** | 502 |  | 0.603 |  |  |  |
| M0 | 3 | Reference |  |  |  |  |
| M1 | 9 | 0.710 (0.130 - 3.886) | 0.693 |  |  |  |
| M2 | 320 | 0.807 (0.200 - 3.259) | 0.763 |  |  |  |
| M4 | 110 | 0.837 (0.204 - 3.443) | 0.805 |  |  |  |
| M5 | 59 | 1.077 (0.259 - 4.479) | 0.919 |  |  |  |
| M7 | 1 | 3.157 (0.285 - 34.961) | 0.349 |  |  |  |
| **Cytogenetic risk stratification** | 502 |  | **< 0.001** |  |  |  |
| Favorable | 91 | Reference |  |  | Reference |  |
| Intermediate | 336 | 1.636 (1.095 - 2.445) | **0.016** |  | 0.943 (0.573 - 1.550) | 0.816 |
| Adverse | 75 | 4.711 (2.988 - 7.428) | **< 0.001** |  | 2.094 (1.094 - 4.009) | **0.026** |
| **2022 ELN risk classification** | 502 |  | **< 0.001** |  |  |  |
| Favorable | 231 | Reference |  |  | Reference |  |
| Intermediate | 130 | 1.797 (1.300 - 2.484) | **< 0.001** |  | 2.552 (1.707 - 3.816) | **< 0.001** |
| Adverse | 141 | 3.156 (2.343 - 4.251) | **< 0.001** |  | 3.867 (2.439 - 6.129) | **< 0.001** |
| **Allo-HSCT** | 502 |  | **< 0.001** |  |  |  |
| No | 308 | Reference |  |  | Reference |  |
| Yes | 194 | 0.409 (0.307 - 0.544) | **< 0.001** |  | 0.266 (0.188 - 0.377) | **< 0.001** |
| **DNMT3A mutations** | 502 |  | 0.078 |  |  |  |
| WT | 393 | Reference |  |  | Reference |  |
| MUT | 109 | 1.300 (0.977 - 1.729) | 0.071 |  | 1.119 (0.800 - 1.565) | 0.512 |
| **TET2 mutations** | 502 |  | **0.017** |  |  |  |
| WT | 426 | Reference |  |  | Reference |  |
| MUT | 76 | 1.509 (1.091 - 2.087) | **0.013** |  | 1.284 (0.903 - 1.825) | 0.164 |
| **IDH1 mutations** | 502 |  | 0.056 |  |  |  |
| WT | 470 | Reference |  |  | Reference |  |
| MUT | 32 | 1.575 (1.016 - 2.441) | **0.042** |  | 1.686 (1.009 - 2.818) | **0.046** |
| **IDH2 mutations** | 502 |  | 0.342 |  |  |  |
| WT | 449 | Reference |  |  |  |  |
| MUT | 53 | 1.209 (0.825 - 1.772) | 0.330 |  |  |  |
| **WT1 mutations** | 502 |  | 0.846 |  |  |  |
| WT | 431 | Reference |  |  |  |  |
| MUT | 71 | 0.965 (0.675 - 1.381) | 0.847 |  |  |  |
| Bone marrow blasts percentage, median (IQR) | 493 | 1.002 (0.996 - 1.008) | 0.551 |  |  |  |
| WBC(×10^9^/L) , median (IQR) | 451 | 1.002 (1.000 - 1.004) | 0.089 |  | 1.002 (1.000 - 1.004) | **0.047** |
| Hemoglobin(g/L), median (IQR) | 449 | 0.998 (0.993 - 1.003) | 0.536 |  |  |  |
| Platelets(×10^9^/L) , median (IQR) | 449 | 1.001 (0.999 - 1.003) | 0.372 |  |  |  |
| age | 500 | 1.018 (1.009 - 1.027) | **< 0.001** |  | 0.999 (0.990 - 1.009) | 0.871 |

WBC, white blood cell; EFS, event-free survival; HSCT, hematopoietic stem cell transplantation; ELN2017, 2017 European LeukemiaNet (ELN) classification guideline.

**Table S7.** Univariate and multivariate analyses of OS in TET2-mutated AML patients from Peking cohort.

| Characteristics | Total(N) | Univariate analysis | |  | Multivariate analysis | |
| --- | --- | --- | --- | --- | --- | --- |
|  |  | Hazard ratio (95% CI) | P value |  | Hazard ratio (95% CI) | P value |
| **Gender** | 76 |  | 0.315 |  |  |  |
| Female | 30 | Reference |  |  |  |  |
| Male | 46 | 1.423 (0.708 - 2.861) | 0.322 |  |  |  |
| **FAB type** | 76 |  | 0.723 |  |  |  |
| M1 | 3 | Reference |  |  |  |  |
| M2 | 49 | 2.070 (0.279 - 15.358) | 0.477 |  |  |  |
| M4 | 18 | 1.420 (0.174 - 11.563) | 0.743 |  |  |  |
| M5 | 5 | 1.419 (0.129 - 15.676) | 0.775 |  |  |  |
| M7 | 1 | 4.695 (0.289 - 76.194) | 0.277 |  |  |  |
| **Number of TET2 mutation sites** | 74 |  | 0.612 |  |  |  |
| 1 | 45 | Reference |  |  |  |  |
| 2 | 27 | 1.429 (0.709 - 2.879) | 0.318 |  |  |  |
| 4 | 2 | 1.242 (0.165 - 9.331) | 0.833 |  |  |  |
| **MRC or MDS** | 76 |  | 0.932 |  |  |  |
| No | 69 | Reference |  |  |  |  |
| Yes | 7 | 0.950 (0.291 - 3.106) | 0.932 |  |  |  |
| **CR1** | 76 |  | **0.002** |  |  |  |
| No | 35 | Reference |  |  | Reference |  |
| Yes | 41 | 0.346 (0.174 - 0.689) | **0.003** |  | 0.286 (0.134 - 0.611) | **0.001** |
| **Cytogenetic risk stratification** | 76 |  | 0.299 |  |  |  |
| Favorable | 9 | Reference |  |  |  |  |
| Intermediate | 57 | 1.630 (0.493 - 5.391) | 0.423 |  |  |  |
| Adverse | 10 | 2.868 (0.714 - 11.521) | 0.137 |  |  |  |
| **2022 ELN risk classification** | 76 |  | 0.487 |  |  |  |
| Favorable | 32 | Reference |  |  |  |  |
| Intermediate | 23 | 1.521 (0.693 - 3.337) | 0.296 |  |  |  |
| Adverse | 21 | 1.506 (0.659 - 3.442) | 0.332 |  |  |  |
| **Allo-HSCT** | 76 |  | **< 0.001** |  |  |  |
| No | 55 | Reference |  |  | Reference |  |
| Yes | 21 | 0.177 (0.054 - 0.581) | **0.004** |  | 0.234 (0.066 - 0.837) | **0.025** |
| **DNMT3A mutations** | 76 |  | 0.904 |  |  |  |
| WT | 57 | Reference |  |  |  |  |
| MUT | 19 | 0.954 (0.446 - 2.043) | 0.904 |  |  |  |
| **IDH1 mutations** | 76 |  | 0.815 |  |  |  |
| WT | 72 | Reference |  |  |  |  |
| MUT | 4 | 1.191 (0.285 - 4.970) | 0.811 |  |  |  |
| **IDH2 mutations** | 76 |  | 0.234 |  |  |  |
| WT | 72 | Reference |  |  |  |  |
| MUT | 4 | 0.360 (0.049 - 2.636) | 0.314 |  |  |  |
| **WT1 mutations** | 76 |  | 0.090 |  |  |  |
| WT | 66 | Reference |  |  | Reference |  |
| MUT | 10 | 0.349 (0.084 - 1.456) | 0.149 |  | 0.431 (0.102 - 1.825) | 0.253 |
| Bone marrow blasts percentage, median (IQR) | 76 | 0.999 (0.985 - 1.013) | 0.912 |  |  |  |
| WBC(×10^9^/L) , median (IQR) | 68 | 1.004 (0.998 - 1.011) | 0.224 |  |  |  |
| Hemoglobin(g/L), median (IQR) | 67 | 1.004 (0.991 - 1.018) | 0.521 |  |  |  |
| Platelets(×10^9^/L) , median (IQR) | 68 | 0.994 (0.987 - 1.002) | 0.162 |  |  |  |
| age | 75 | 1.022 (0.999 - 1.046) | 0.057 |  | 1.021 (0.993 - 1.051) | 0.141 |
| TET2 mutation frequency | 74 | 0.770 (0.111 - 5.365) | 0.792 |  |  |  |
| WT1 expression level at primary diagnosis | 71 | 1.011 (0.999-1.022) | 0.062 |  | 1.013 (1.002 - 1.025) | **0.017** |

WBC, white blood cell; MRC, myelodysplasia-related changes; MDS, myelodysplastic syndrome; CR1, complete remission in phase 1 chemotherapy.





**Figure S1.** The frequency (a) and types (b) of TET2 mutations in AML patients in the Beat AML database.


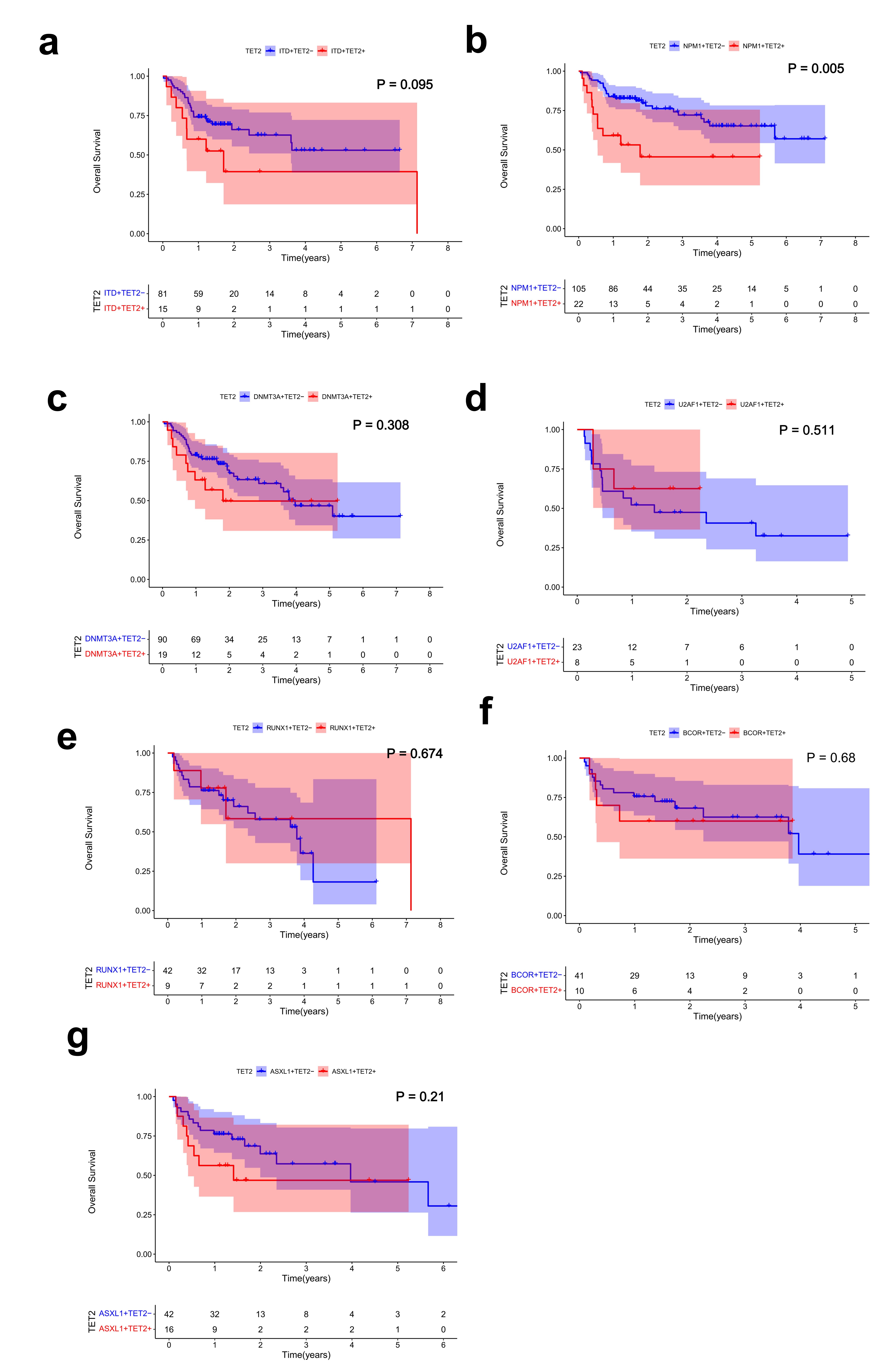


**Figure S2. Prognostic significance of TET2 mutations in subgroups of AML with distinct mutational profiles.** (a) Kaplan-Meier survival analysis of OS in FLT-ITD+ AML patients. (b) Kaplan-Meier survival analysis of OS in NPM1+ AML patients. (c) Kaplan-Meier survival analysis of OS in DNMT3A+ AML patients. (d) Kaplan-Meier survival analysis of OS in U2AF1+ AML patients. (e) Kaplan-Meier survival analysis of OS in RUNX1+ AML patients. (f) Kaplan-Meier survival analysis of OS in BCOR+ AML patients. (g) Kaplan-Meier survival analysis of OS in ASXL1+ AML patients.


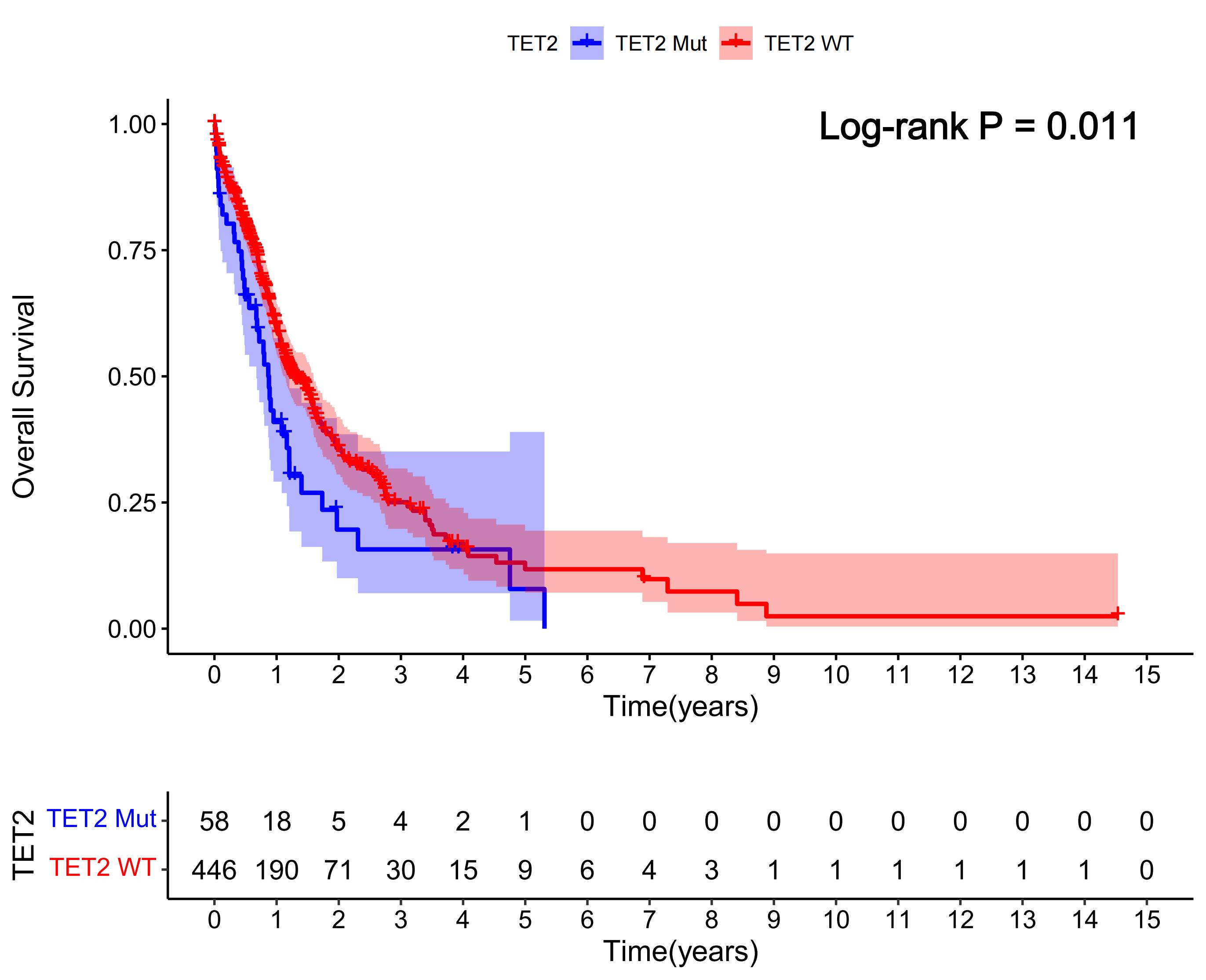


**Figure S3.** Kaplan-Meier survival analysis of OS according to TET2 mutation status in the Beat AML cohort.

**
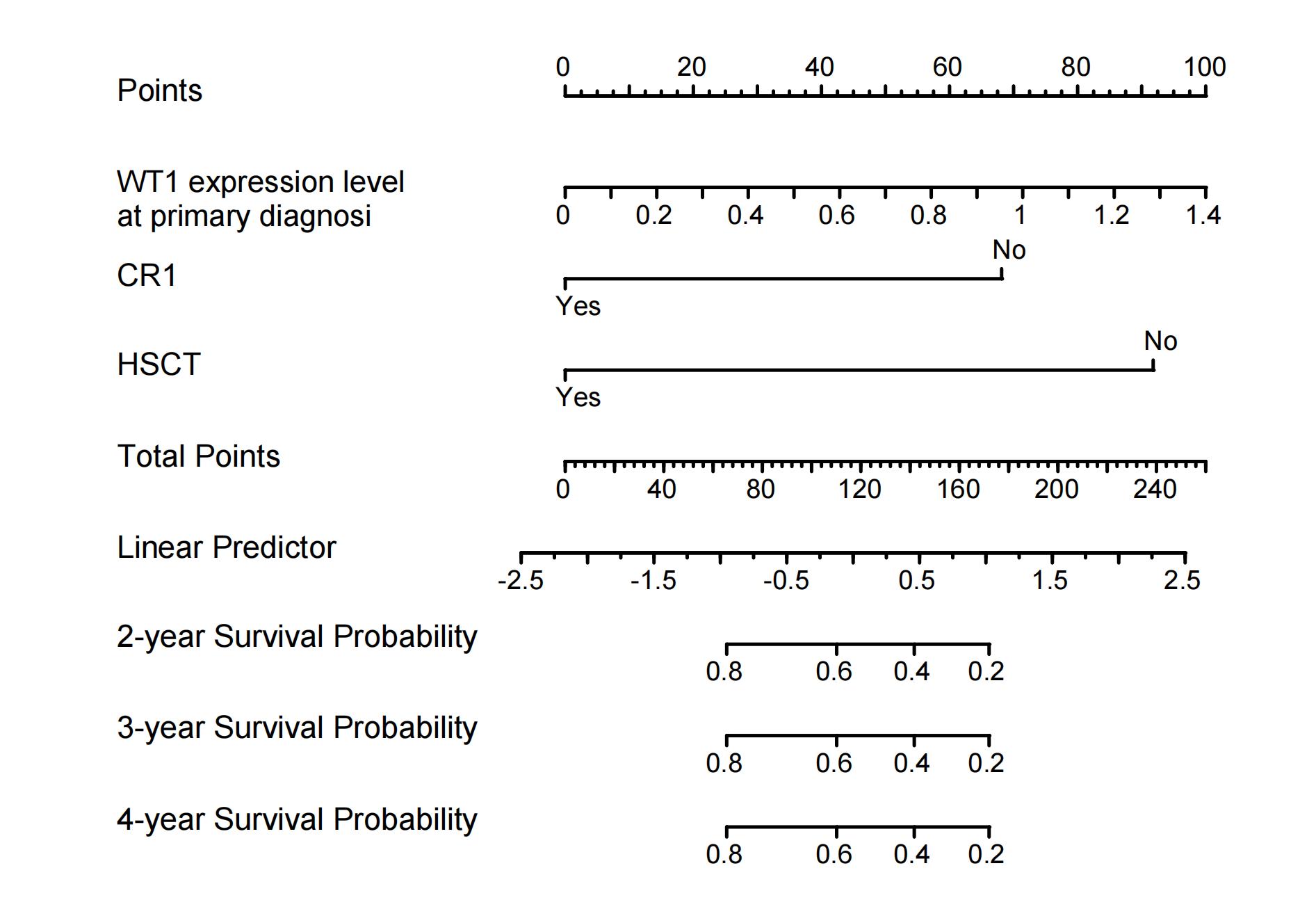
**

**Figure S4.** Nomogram model combing 3 characteristics for predicting survival probability in 76 AML patients of Peking cohort with TET2 mutation (HSCT, CR1, WT1 expression level at primary diagnosis). The cumulative score is positioned along the axis of total points, and a line is extended vertically to intersect with the survival axes, indicating the probability of 2-year, 3-year, or 4-year overall survival.





**Figure S5.** (a)GSEA enrichment analysis of KEGG signaling pathways between TET2+ AML and TET2- AML (Calcium signaling pathway: NES=1.45, p-value=0.005; Chemokine signaling pathway: NES=1.59, p-value=0.0008; Linoleic acid metabolism: NES=-1.74, p-value=0.007; Melanogenesis: NES=1.53, p-value=0.008; Nod like receptor signaling pathway: NES=1.64, p-value=0.003). (b) Venn diagram for the overlapping genes (TET2+ AML patients VS. healthy donors and TET2- AML patients VS. healthy donors).


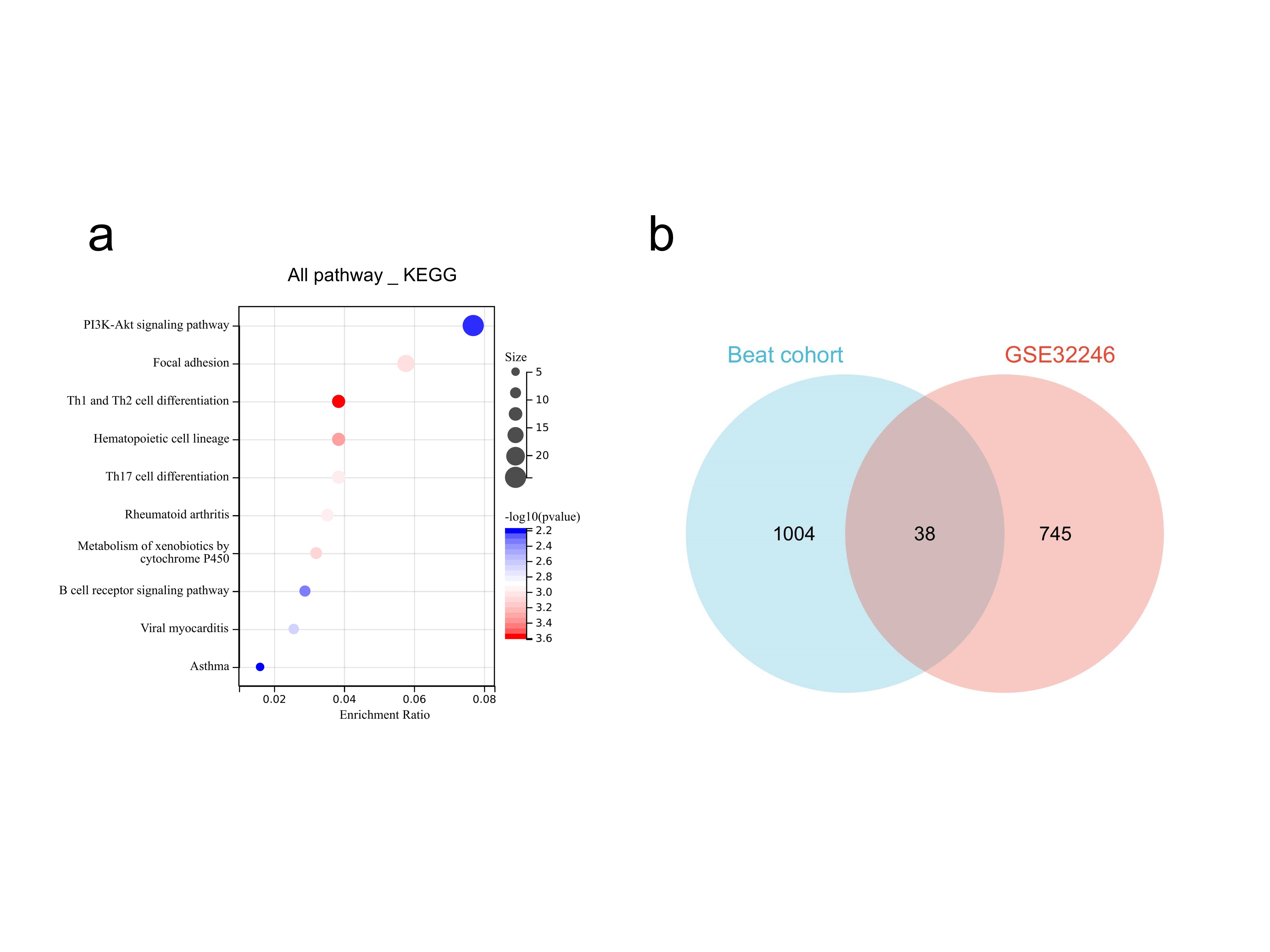


**Figure S6.** (a) KEGG Pathway Analysis of Differentially Expressed Genes (DEGs) in the GSE32246 dataset. (b) Venn Diagram illustrating the overlap of DEGs between the Beat AML and the GSE32246 dataset.
